# Characterization of *α*-Glucosidase Inhibitor/Cyclodextrin Complex Prepared by Freeze-Drying

**DOI:** 10.1155/2018/3202719

**Published:** 2018-05-07

**Authors:** Yutaka Inoue, Sachie Narumi, Akiho Mitsumori, Isamu Murata, Ikuo Kanamoto

**Affiliations:** Laboratory of Drug Safety Management, Faculty of Pharmacy and Pharmaceutical Sciences, Josai University, 1-1 Keyakidai, Sakado-shi, Saitama 3500295, Japan

## Abstract

Miglitol (MT) is an *α*-glucosidase inhibitor with a postmeal blood glucose level lowering effect that is used to treat type 2 diabetes. In addition, *α*-cyclodextrin (*α*CD) has been reported to inhibit increases in postmeal blood glucose. The aim of this study was to prepare a freeze-dried product (FD) composed of MT and *α*CD or *γ*CD (molar ratio of MT/*α*CD = 1/1, MT/*γ*CD = 1/1) and to evaluate the physicochemical properties and biological activity of the FD. The PXRD profile of FD exhibited a halo pattern, and characteristic peaks derived from MT, *α*CD, and *γ*CD were not observed. The TG-DTA results for FD indicated an increased weight loss temperature and the absence of an endothermic peak for MT. The NIR absorption spectrum measurement suggested an intermolecular interaction between MT and *α*CD or *γ*CD in the FD. ^1^H-^1^H NOESY NMR spectroscopy (D_2_O) revealed an intermolecular interaction in the FD. The results of the *α*-glucosidase activity inhibition test and the *α*-amylase activity inhibition test indicated that the FD exhibited the same inhibition rate as MT alone and the effects of MT were not altered by the freeze-drying method.

## 1. Introduction

Recently, the incidence of diabetes has increased in developed countries. This has been attributed to smoking, an inadequate diet, and lack of exercise, and diabetes has become a major cause of the increasing death rate [1]. According to the International Diabetes Federation report, the world diabetes population continues to increase and in 2015, 415 million people were suffering from diabetes. For this reason, the treatment of prediabetes by controlling blood glucose at levels lower than that required for a diabetes diagnosis to prevent the development of full blown diabetes has been gaining attention.

Cyclodextrins (CDs) are cyclic dextrins currently used as food additives. Recently, *α*CD was reported to lower blood sugar levels. Specifically, *α*CD inhibited pancreatic amylase activity [2] and suppressed increases in blood sugar [3]. In addition, CDs solubilize, stabilize, and mask drugs by inclusion in a complex. For example, formulation of an inclusion complex made of itraconazole, a sparingly soluble antifungal agent, and hydroxypropyl-*β*CD [4] increased the solubilization of the drug. Additionally, formulation of an inclusion complex made of limaprost, a prostaglandin E_1_ derivative that is unstable to moisture, and *α*-cyclodextrin [5] increased the drug's stability. Further, masking a drug with cyclodextrin decreased the bitter taste of the drug [6].

The currently available *α*-glucosidase inhibitors include acarbose (AB), voglibose (VB), and miglitol (MT). AB, VB, and MT are used as therapeutic agents for type 2 diabetes. Among them, AB inhibits *α*-amylase and *α*-glucosidase activity. Additionally, VB and MT are widely used clinically because their *α*-glucosidase inhibitory action is stronger than that of AB. In particular, MT is different from the other two drugs and is quickly absorbed in the upper part of the small intestine. This characteristic suppresses the rise in blood glucose level starting 30 min after a meal and continuing until 2 h after a meal. MT is particularly effective at inhibiting the increase in blood glucose at 1 h after meal.

Therefore, this study focused on MT and aimed to develop a formulation that would provide an additive synergistic suppressive effect on blood glucose elevation by forming a complex with *α*CD. In this report, inclusion complexes with MT were prepared using *α*CD and *γ*CD. In addition, the addictive effect of *α*CD and *γ*CD with *α*-glucosidase inhibitors was evaluated.

## 2. Materials and Methods

### 2.1. Materials

**Figure 1:**
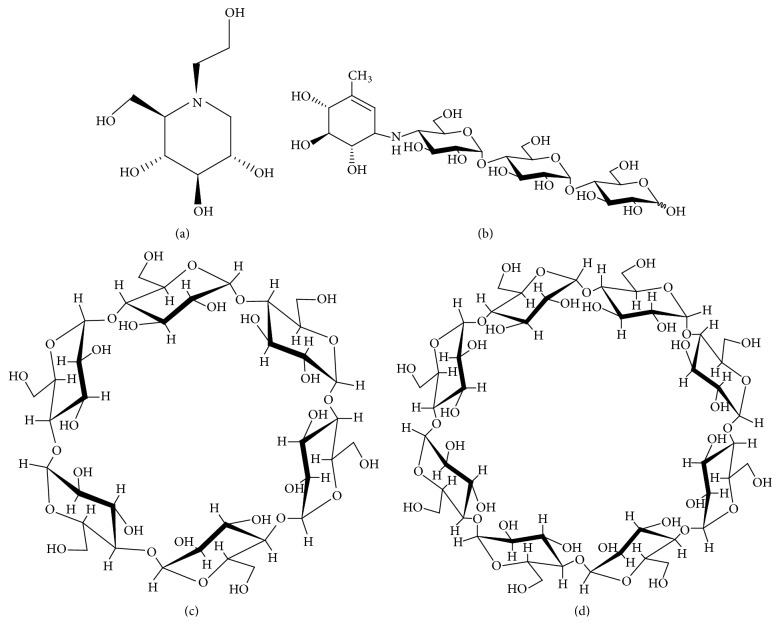
Chemical structures of (a) miglitol, (b) acarbose, (c) *α*CD, (d) *γ*CD.

*α*CD and *γ*CD were donated by Cyclo Chem Co., Ltd. (Tokyo, Japan) and stored at a temperature of 40°C and relative humidity of 82% for 7 days before use. MT was donated by SANWA KAGAKU KENKYUSHO Co., Ltd (Figure 1). All other chemicals were of analytical grade and obtained from Wako Pure Chemical Industries., Ltd.

### 2.2. Methods

#### 2.2.1. Preparation of Physical Mixture

A physical mixture (PM) of MT/*α*CD and a PM of MT/*γ*CD at a molar ratio of 1/1 was prepared by blending the two compounds for 1 min using a vortex mixer.

#### 2.2.2. Preparation of Freeze-Dried Product

Complexes were formed using a freeze-drying method. The mixtures prepared with MT/*α*CD = 1/1 and MT/*γ*CD = 1/1 were dissolved in 100 mL of purified water and prefrozen at −30°C or lower. Thereafter, the prefrozen product was freeze-dried in a vacuum freeze-dryer (FZ-6 model manufactured by ALS Co., Ltd.) for 24 h to produce the freeze-dried product (FD).

#### 2.2.3. Powder X-Ray Diffraction (PXRD)

Powder X-ray diffraction was performed using an X-ray diffractometer (MiniFlex II, Rigaku) with Cu radiation, a scan range of 2*θ* = 5–40°, and a scan rate of 4°/min. The intensities of diffraction were measured using a NaI scintillation counter coupled to a discriminator.

#### 2.2.4. Thermogravimetry/Differential Thermal Analyzer

The thermal behavior of the samples was analyzed using a thermogravimetry/differential thermal analyzer (Thermo plus Evo, Rigaku) under a nitrogen flow rate of 200 mL/min. The samples were heated at a scanning rate of 5.0°C/min from 30°C to 350°C.

#### 2.2.5. Near Infrared (NIR) Absorption Spectrometry

Each sample was filled in with a Fourier transform type near infrared spectrometer (Buchi N-500: Nippon Buchi), under conditions of a measurement wavelength of 10000–4000 cm^−1^, a measuring time of 8 s, and a measuring temperature of 25°C with an interval of 1 nm of optical path.

#### 2.2.6. ^1^H-^1^H Nuclear Overhauser Effect Spectroscopy (NOESY) NMR Spectroscopy

Two-dimensional (2D) NOESY NMR spectroscopy and selective 1D-NMR spectroscopy were performed using an NMR spectrometer (Varian NMR System 700NB, Agilent) with a cold probe operating at 699.6 MHz and a D_2_O solution. The measurement conditions were as follows: an acquisition time of 7.0 *μ*s, a pulse width of 90°, a relaxation delay of 0.267 s, a mixing time of 4.500 s, a fixed delay of 1.500 s, and a temperature of 298 K.

#### 2.2.7. *α*-Glucosidase Activity Inhibition Test

One hundred microliters of each test solution (MT, *α*CD, *γ*CD, PM (MT/*α*CD), PM (MT/*γ*CD), FD (MT/*α*CD), and FD (MT/*γ*CD)) and 600 *μ*L of 0.1 mol/L maleate buffer (pH 6.0) were added to 100 *μ*L of a substrate solution (1% maltose). Thereafter, 200 *μ*L of *α*-glucosidase solution (active solution, inactive solution) was added and the solution was incubated for 60 min at 37°C to allow an *α*-glucosidase reaction. The reaction was stopped by heating the solution at 100°C for 15 min. Thereafter, 10 *μ*L of the reaction solution was dispensed into a 96-well microplate and 200 *μ*L of Gluest CII-Test Wako, coloring reagent (Wako Pure Chemical Industries, Ltd.), was added and the solution was incubated for 5 min at 37°C. After incubation, the absorbance was measured using a microplate reader (Spectra Max 190®, Molecular Devices Japan Ltd.) at a wavelength of 505 nm. To 2.0 g of rat acetone powder (intestinal acetone powder from rats, Sigma-Aldrich), 20 mL of 0.1 mol/L maleic acid buffer solution (pH 6.0) was added and the mixture was homogenized for 1 min. Thereafter, the mixture was centrifuged and the supernatant was used as an *α*-glucosidase active solution. In addition, this solution was heated at 100°C for 15 min to obtain an inert liquid. The *α*-glucosidase active solution was used as a positive control and the inactive solution was used as a negative control. For the test solution, acarbose was used as the control agent. The IC_50_ value of acarbose was calculated from the dose-response curves (not given in this paper). The results indicated that the IC_50_ value of acarbose was 87 *μ*g/mL, which was close to the value reported by Yang et al. [7]. Therefore, the concentration of MT was set by taking the ratio between 100 mg of one dose of acarbose and 50 mg of one dose of MT. The concentrations of PM (MT/*α*CD), PM (MT/*γ*CD), FD (MT/*α*CD), and FD (MT/*γ*CD) were adjusted to be equal to that of MT alone. In addition, the concentration of *α*CD was adjusted to be equal to that of PM (MT/*α*CD) and FD (MT/*α*CD), and the concentration of *γ*CD was equal to that of PM (MT/*γ*CD) and FD (MT/*γ*CD).

The *α*-glucosidase activity inhibition rate was calculated using the following formula:

(1) $$\begin{array}{c} \alpha \text{‐glucosidase inhibition}\%=100-\frac{\left(C-D\right)}{\left(A-B\right)}\times 100, \end{array}$$

where *A* is the glucose concentration in the positive control, *B* is the glucose concentration in the negative control, *C* is the glucose concentration obtained by adding the test solution to the positive control, and *D* is the glucose concentration obtained by adding the test solution to the negative control.

#### 2.2.8. *α*-Amylase Activity Inhibition Test

Fifty microliters of each test solution (MT, *α*CD, *γ*CD, PM (MT/*α*CD), PM (MT/*γ*CD), FD (MT/*α*CD), and FD (MT/*γ*CD)) and 400 *μ*L of phosphate buffered saline (pH 7.4) were added to 450 *μ*L of a substrate solution (0.5% starch aqueous solution). Thereafter, 100 *μ*L of *α*-amylase solution (active solution, inactive solution) was added and the mixture was incubated for 20 min at 37°C to perform an *α*-amylase reaction. Thereafter, 500 *μ*L of DSN reagent (1% 3,5-dinitrosalicylic acid and 12% potassium sodium tartrate in 0.4 mol/L NaOH) was added to the incubated solution and the mixture was heated at 100°C for 15 min to stop the reaction. The reaction solution (200 *μ*L) was then dispensed into a 96-well microplate and the absorbance was ELISA-assayed at a wavelength of 540 nm using a microplate reader (Spectra Max 190, Molecular Devices Japan KK). To 16.5 mg of porcine pancreatic-derived *α*-amylase (*α*-amylase from porcine pancreas, 19.5 unite amylase/mg, SIGMA), 100 mL of phosphate buffered saline was added. After ultrasonic treatment for 10 min, the mixture was centrifuged and the supernatant was used as an *α*-amylase active solution. In addition, this solution was heated at 100°C for 15 min to obtain an inert liquid. The *α*-amylase active solution was used as a positive control and the inactive solution as a negative control. For the test solution, acarbose was used as a control agent. The IC_50_ value of acarbose was calculated from the dose-response curves (not given in this paper). The results indicated that the IC_50_ value of acarbose was 20 *μ*g/mL, which was close to the value reported by Wickramaratne et al. [8]. Therefore, the concentration of MT was set by taking the ratio between 100 mg of one dose of acarbose and 50 mg of one dose of MT. The concentrations of PM (MT/*α*CD), PM (MT/*γ*CD), FD (MT/*α*CD), and FD (MT/*γ*CD) were adjusted to be equal to that of MT alone. In addition, the concentration of *α*CD was adjusted to be equal to that of PM (MT/*α*CD) and FD (MT/*α*CD), and the concentration of *γ*CD was adjusted to be equal to that of PM (MT/*γ*CD) and FD (MT/*γ*CD).

The *α*-amylase activity inhibition rate was calculated using the following formula:

(2) $$\begin{array}{c} \alpha \text{‐Amylase Inhibition}\%=100-\frac{\left(C-D\right)}{\left(A-B\right)}\times 100, \end{array}$$

where *A* is the glucose concentration in the positive control, *B* is the glucose concentration in the negative control, *C* is the glucose concentration obtained by adding the test solution to the positive control, and *D* is the glucose concentration obtained by adding the test solution to the negative control.

## 3. Results and Discussion

### 3.1. PXRD Measurement

**Figure 2:**
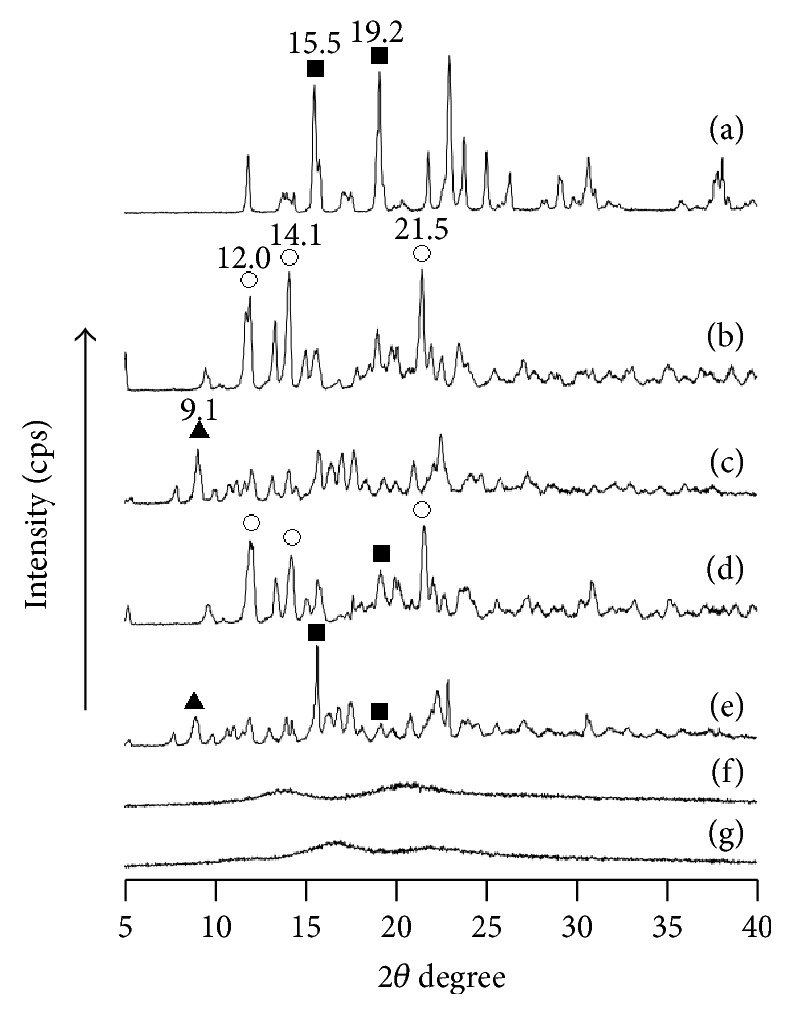
PXRD patterns of MT/*α*CD and MT/*γ*CD systems. (a) MT intact, (b) *α*CD, (c) *γ*CD, (d) PM (MT/*α*CD = 1/1), (e) PM (MT/*γ*CD = 1/1), (f) FD (MT/*α*CD = 1/1), (g) FD (MT/*γ* CD = 1/1). ■: MT original, ○: *α*CD original, ▲: *γ* CD original.

PXRD was performed to examine the crystalline state of MT and CDs in the FD (MT/*α*CD) and FD (MT/*γ*CD) preparations (Figure 2). Intact MT exhibited characteristic diffraction peaks at 2*θ* = 15.5° and 19.2°. *α*CD exhibited characteristic diffraction peaks at 2*θ* = 12.0°, 14.1°, 21.52°. The PM (MT/*α*CD) exhibited diffraction peaks derived from *α*CD at 2*θ* = 12.0°, 14.1°, 21.5° and MT at 2*θ* = 19.2°. However, FD (MT/*α*CD) exhibited a halo pattern. *γ*CD exhibited a characteristic diffraction peak at 2*θ* = 9.1°. The PM (MT/*γ*CD) exhibited diffraction peaks derived from *γ*CD at 2*θ* = 9.10° and MT at 2*θ* = 15.5° and 19.16°. However, FD (MT/*γ*CD) exhibited a halo pattern similar to that of FD (MT/*α*CD) and an amorphous state was observed. An amorphous state observed during the PXRD measurement of a solid dispersion of CD and a guest molecule suggests that there is a possibility that CD and the guest molecule formed an inclusion complex [9, 10]. From this fact, we presumed that some interaction occurred in the crystalline state during the freeze-drying preparation of FD (MT/*α*CD) and FD (MT/*γ*CD).

### 3.2. TG-DTA

**Figure 3:**
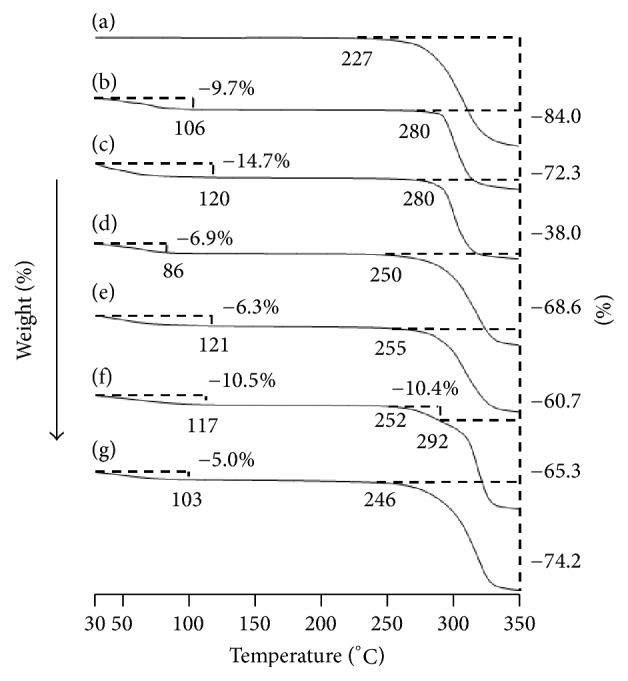
TG curves of MT/*α*CD and MT/*γ*CD systems. (a) MT, (b) *α*CD, (c) *γ*CD, (d) PM (MT/*α*CD = 1/1), (e) PM (MT/*γ*CD = 1/1), (f) FD (MT/*α*CD = 1/1), (g) FD (MT/*γ*CD = 1/1).

**Figure 4:**
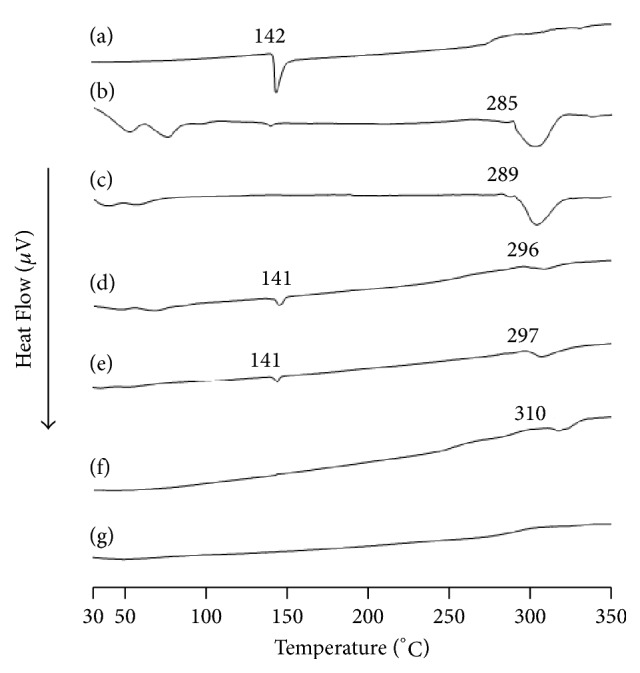
DTA curves of MT/*α*CD and MT/*γ*CD systems. (a) MT, (b) *α*CD, (c) *γ*CD, (d) PM (MT/*α*CD = 1/1), (e) PM (MT/*γ*CD = 1/1), (f) FD (MT/*α*CD = 1/1), (g) FD (MT/*γ*CD = 1/1).

An amorphous state was observed in FD (MT/*α*CD) and FD (MT/*γ*CD) during the PXRD measurement. Therefore, a TG-DTA measurement was performed to confirm the thermal behavior (Figures 3 and 4). For the intact MT, thermal weight reduction was observed around 227°C. Conversely, a thermal weight decrease was observed at 252°C and 246°C for FD (MT/*α*CD) and FD (MT/*γ*CD). In addition, in the intact MT, an endothermic peak owing to the melting point of MT was confirmed around 142°C. Even in the PM (MT/*α*CD) and PM (MT/*γ*CD), an endothermic peak owing to the melting point of MT was observed around 141°C. However, an endothermic peak owing to the melting point of MT was not observed in FD (MT/*α*CD) and FD (MT/*γ*CD). The increase in the thermal weight reduction temperature and the disappearance of the endothermic peak of the guest molecule indicated that an inclusion complex made of CD and the guest molecule was formed [11, 12]. These changes in thermal behavior suggested intermolecular interactions in MT/*α*CD and MT/*γ*CD lyophilizates. Incidentally, there was no change in the results of PXRD and TG-DTA measurement after a month of preparation of FD (MT/*α*CD) and FD (MT/*γ*CD) (not shown data). Therefore, the freeze-dried product (MT/*α*CD and MT/*γ*CD) presume is stable.

### 3.3. NIR

**Figure 5:**
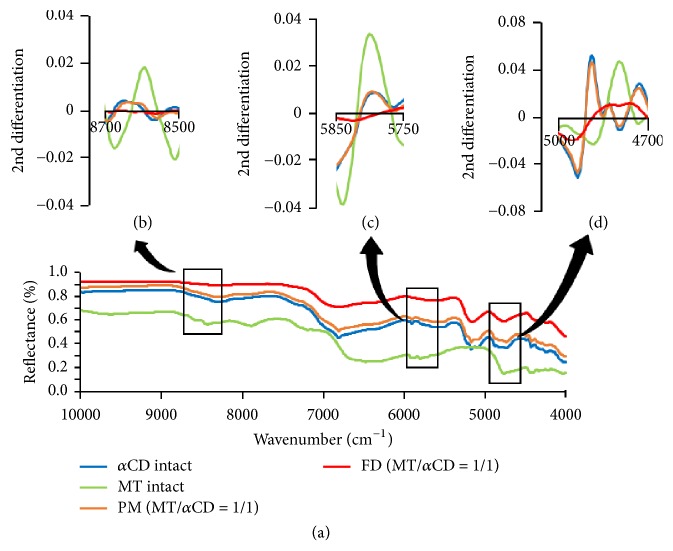
NIR absorption spectra of MT/*α*CD systems and second-differentiation NIR absorption spectra of MT/*α*CD systems ((b) alkyl group, (c) alkyl group, and (d) hydroxy group).

**Figure 6:**
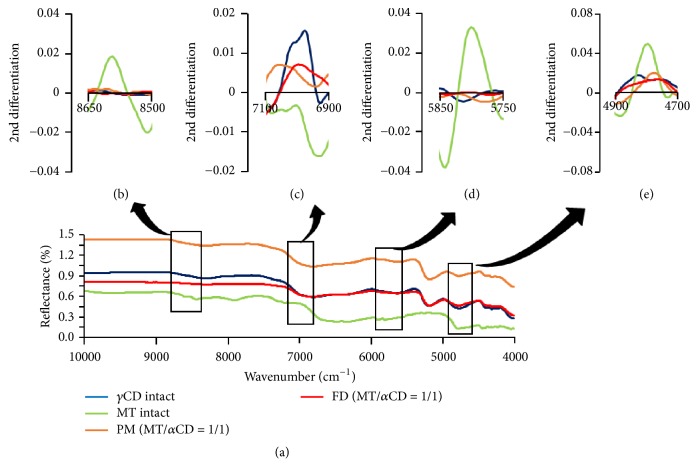
NIR absorption spectra of MT/*γ*CD systems and second-differentiation NIR absorption spectra of MT/*γ*CD systems ((b) alkyl group of MT, (c) hydroxy group of *γ*CD, (d) alkyl group of MT, and (e) hydroxy group of MT).

PXRD measurement and TG-DTA measurement suggested inclusion complex formation in FD (MT/*α*CD) and FD (MT/*γ*CD). Therefore, to verify the detailed intermolecular interaction, the NIR absorption spectrum was measured (Figures 5 and 6).

The results of the NIR measurement indicated that the -OH group was observed around 4800 cm^−1^ [13] and 6660 cm^−1^–7140 cm^−1^ [14]. In addition, the -CH_2_ group was observed around 5800 cm^−1^ [15] and 8145 cm^−1^–9153 cm^−1^ [16]. From the results of the NIR measurement, a peak derived from an -OH group appeared around 4800 cm^−1^ and that from a -CH_2_ group appeared around 5800 cm^−1^ and 8600 cm^−1^ for MT. In *α*CD alone, a peak derived from an -OH group appeared around 4900 cm^−1^ (Figure 5(a)). In the second derivative spectrum, the peak of the -CH_2_ group around 8600 cm^−1^ (Figure 5(b)) and 5800 cm^−1^ (Figure 5(c)) of MT was broadened in FD (MT/*α*CD). In addition, the peak derived from the -OH group around 4800 cm^−1^ of MT and the -OH group around 4900 cm^−1^ of *α*CD was broadened (Figure 5(d)). A broadened or shifted peak in the NIR measurement indicated that there were intermolecular interactions between the drug and functional group [17]. This suggested that there were intermolecular interactions between the -CH_2_ group and -OH group of MT and the -OH group of *α*CD in FD (MT/*α*CD).

In addition, in *γ*CD alone, a peak derived from the -OH group appeared around 7000 cm^−1^ (Figure 6(a)). In the results of the second derivative spectrum, the peak of the -OH group around 4800 cm^−1^ (Figure 6(e)) and the peak of -CH_2_ group around 8600 cm^−1^ (Figure 6(b)) and 5800 cm^−1^ (Figure 5(d)) for MT were broadened in FD (MT/*γ*CD).

In addition, the peak derived from the -OH group around 7000 cm^−1^ of *γ*CD shifted to the higher wave number (Figure 6(c)). This suggested that there were intermolecular interactions between the -CH_2_ group and the -OH group in MT and the -OH group in *γ*CD in FD (MT/*γ*CD).

Miglitol has a -CH_2_ group at the 6th position in its side chain and at a cyclic structure. Therefore, in the NIR measurement, it is impossible to determine whether the -CH_2_ group is of the cyclic structure or the 6th position in the side chain. Therefore, detailed examination by 2D-NMR measurement is necessary.

### 3.4. ^1^H-^1^H NOESY NMR Spectroscopy

**Figure 7:**
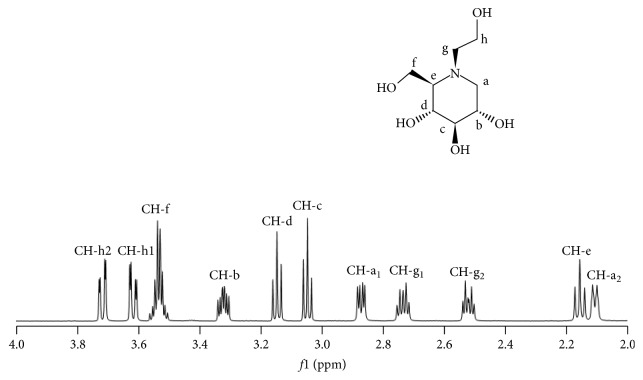
Proton-NOESY NMR spectra of MT in D_2_O.

**Figure 8:**
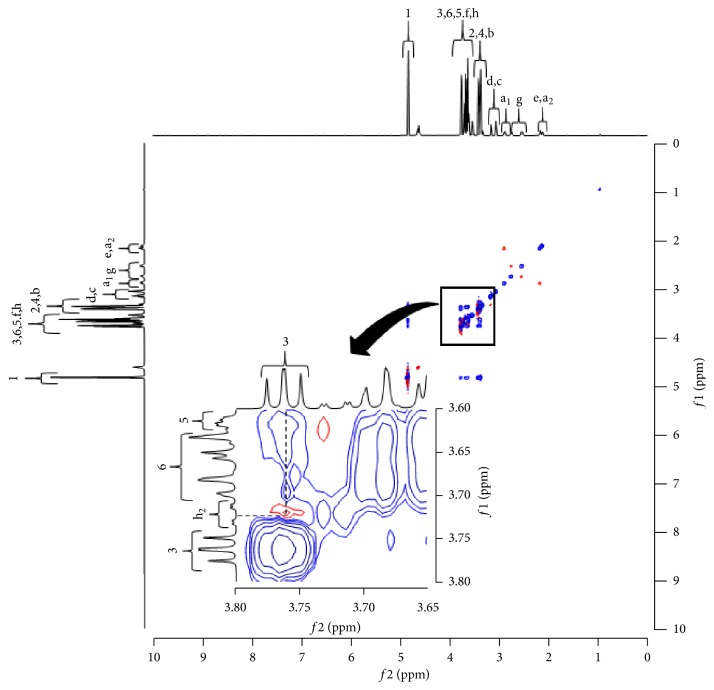
^1^H-^1^H -NOESY NMR spectra of MT/*α*CD system.

**Figure 9:**
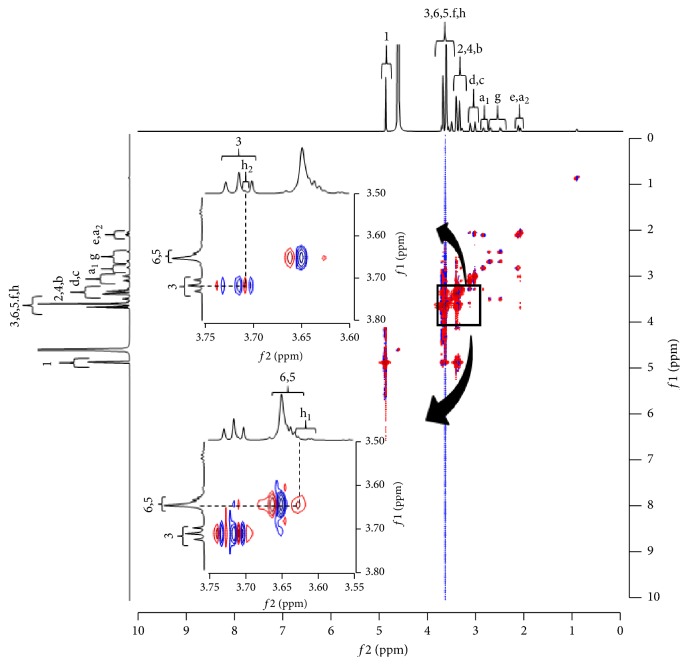
^1^H-^1^H -NOESY NMR spectra of MT/*γ*CD system.

NIR measurement indicated an intermolecular interaction between the -CH_2_ and -OH group of MT and the -OH group of *α*CD and between the -CH_2_ and -OH group of MT and the -OH group of *γ*CD. Therefore, the detailed intermolecular interaction was further evaluated. First, ^1^H-NMR measurement of MT alone was conducted to confirm the attributions of MT. The results indicated that the -CH (h) of MT exhibited two peaks at 3.62 ppm (h_1_) and 3.72 ppm (h_2_) (Figure 7). Thereafter, ^1^H-^1^H NOESY NMR spectroscopy measurement was performed (Figures 8 and 9). In FD (MT/*α*CD), cross peaks were observed between the peak for the -CH (h_2_: 3.72 ppm) in MT and the H-3 (3.76 ppm) protons in *α*CD (Figure 8). In general, it has been reported that by observing H-3, 5, and 6 protons of the CD cavity and protons of the guest molecule using ^1^H-^1^H-NMR, the structure of the inclusion complex can be elucidated [18]. In addition, from this result, a cross peak between H-3 showing the wide edge of CD and the -CH (h_2_: 3.72 ppm) in MT was observed in FD (MT/*α*CD). This suggested that the inclusion complex made of *α*CD and MT was in a form covering the alkyl group of MT from the cavity of a wide edge of CD with respect to the alkyl group of the -CH (h) of MT.

Additionally, in FD (MT/*γ*CD), cross peaks were observed between the peak for -CH (h_1_: 3.62 ppm, h_2_: 3.72 ppm) of MT and the H-3 (3.72 ppm) and H-6 (3.65 ppm) protons of *γ*CD (Figure 9). From this result, a cross peak between H-3 showing the wide edge of CD and -CH (h_2_: 3.72 ppm) of MT was observed in FD (MT/*γ*CD). This suggested that the inclusion complex made of *γ*CD and MT included a form covering the alkyl group of MT from the cavity of a wide edge of CD with respect to the alkyl group of the -CH (h) in MT. This confirmed that the inclusion complex with MT was similar between that with *α*CD and that with *γ*CD with different cavity diameters.

### 3.5. *α*-Glucosidase Activity Inhibition Test

Inclusion complex formation was confirmed for FD (MT/*α*CD) and FD (MT/*γ*CD). Because deterioration of bioactivity is an area of concern for inclusion preparations, the MT in inclusion complexes was tested for *α*-glucosidase activity (Table 1). For intact MT, a 51.2% inhibition of *α*-glucosidase activity was observed. In addition, FD (MT/*α*CD) and FD (MT/*γ*CD) exhibited inhibition rates of 56.2% and 56.6%, respectively, which was similar to the inhibition rates observed with intact MT. Inhibition of *α*-glucosidase activity was not observed in *α*CD or *γ*CD. This indicated that the *α*-glucosidase inhibitory activity depended on the concentration of MT and confirmed that the *α*-glucosidase inhibitory activity of MT in the inclusion complex was sufficiently maintained.

### 3.6. *α*-Amylase Activity Inhibition Test

*α*CD has been reported to inhibit *α*-amylase activity. To confirm the influence on the inhibition of *α*-amylase activity by the formation of these complexes, an *α*-amylase activity inhibition test was conducted (Table 1). Intact MT did not inhibit *α*-amylase activity. Conversely, *α*CD and *γ*CD exhibited inhibition rates of 4.41% and 3.10%. In addition, FD (MT/*α*CD) and FD (MT/*γ*CD) exhibited inhibition rates of 3.21% and 1.29%, which were almost equal to the inhibition rates observed with *α*CD and *γ*CD alone. This was due to inhibition of *α*-amylase activity by both *α*CD and *γ*CD and indicated that the *α*-amylase inhibitory activity of *α*CD and *γ*CD in the prepared complex was sufficiently maintained. The dose of CD used to prepare the FD was very small compared with the dose reported to affect the inhibition of *α*-amylase activity [19]. Therefore, the *α*-amylase activity rate was very low. In the future, it will be necessary to confirm these results under different conditions. Further investigation should evaluate the effects on blood sugar in animal studies using these preparations.

In this study, MT/*α*CD and MT/*γ*CD were evaluated, but a preparation containing MT/*β*CD should also be evaluated. However, MT/*β*CD is not as easily prepared by freeze-drying as MT/*α*CD and MT/*γ*CD. The cavity of *β*CD is hydrophobic compared to *α*CD and *γ*CD. On the other hand, MT of a drug used as a guest molecule in this study is a water-soluble drug. Therefore, MT/*β*CD inclusion complex might not be confirmed. In the future, we plan to develop a better preparation method.

## 4. Conclusion

In this study, we prepared MT/*α*CD complex and MT/*γ*CD complex using a freeze-drying method. In addition, it was confirmed that the structure of the inclusion complex differed depending on the size of the CD ring. Furthermore, we found that there was no influence on the inhibition of *α*-glucosidase or *α*-amylase activity depending on the preparation process used for the FD.

## Figures and Tables

**Table 1:** Inhibitory activities *α*-glucosidase and *α*-amylase.

	Percentage of inhibition	
	*α*-Glucosidase	*α*-Amylase
MT	51.2 ± 7.3	—
*α*CD	—	4.4 ± 2.5
*γ*CD	—	3.1 ± 1.1
PM (MT/*α*CD)	51.5 ± 10.2	0.1 ± 0.8
PM (MT/*γ*CD)	56.2 ± 6.8	3.2 ± 2.2
FD (MT/*α*CD)	49.9 ± 7.4	2.8 ± 1.1
FD (MT/*γ*CD)	56.6 ± 4.9	1.3 ± 2.6

Values are expressed as mean ± standard deviation (*n* = 3). Each data value showed statistically significant difference at *p* value < 0.05 using Turkey's multiple comparison tests.
